# Supplementary material for: Asynchronous magnetic resonance elastography: Shear wave speed reconstruction using noise correlation of incoherent waves
Source: Magn Reson Med. 2022 Oct 27;89(3):990–1001. doi: 10.1002/mrm.29502 (PMC9792433; doi:10.1002/mrm.29502)
Supplement: Supplementary file 1 — DATA S1 MATLAB code used to generate the numerical simulations of Figure 1 [file MRM-89-990-s002.zip › k-Wave/helpfiles/kWaveTransducer.html]

kWaveTransducer :: Functions (k-Wave)


# kWaveTransducer

Class definition for k-Wave linear array transducer.

## Syntax

```
transducer = kWaveTransducer(kgrid, settings)
```

## Description

`kWaveTransducer` is the class definition for k-Wave transducer objects which can be substituted for the source or sensor inputs when using `kspaceFirstOrder3D`. For additional information, see the Defining An Ultrasound Transducer Example.

Note: This function will not work with older versions of MATLAB in which custom class definitions are not supported.

## Inputs

|  |  |
| --- | --- |
| `kgrid` | k-Wave grid object returned by `kWaveGrid` |
| `settings` | input structure used to define the properties of the transducer (see below) |

The following parameters can be appended as fields to the `settings` input structure. These parameters are fixed when the transducer is initialised (they cannot be changed without re-creating the transducer). All the settings are optional and are given their default values if not defined by the user.

| Property | Description | Default |
| --- | --- | --- |
| `number_elements` | total number of transducer elements | `128` |
| `element_width` | width of each element in grid points | `1` |
| `element_length` | length of each element in grid points | `10` |
| `element_spacing` | spacing (kerf width) between the transducer elements in grid points | `0` |
| `position` | position of the corner of the transducer within the grid in grid points | `[1, 1, 1]` |
| `radius` | radius of curvature of the transducer [m] (currently only `inf` is supported) | `inf` |
| `input_signal` | signal used to drive the ultrasound transducer (where the sampling rate is defined by `kgrid.dt` or `kgrid.t_array`) | `[]` |

The following parameters can also be appended as fields to the `settings` input structure, and can additionally be modified after the transducer has been initialised.

| Property | Description | Default |
| --- | --- | --- |
| `active_elements` | transducer elements that are currently active elements | *(all elements)* |
| `beamforming_delay_offset` | beamforming delay offset (used to force beamforming delays to be positive) | `'auto'` |
| `elevation_focus_distance` | focus depth in the elevation direction [m] | `inf` |
| `focus_distance` | focus distance used to calculate beamforming delays [m] | `inf` |
| `receive_apodization` | receive apodization - can be set to any of the window shapes supported by `getWin`, or given as a vector the same length as `number_elements` | `'Rectangular'` |
| `sound_speed` | sound speed used to calculate beamforming delays [m/s] | `1540` |
| `steering_angle` | steering angle used to calculate beamforming delays [deg] | `0` |
| `transmit_apodization` | transmit apodization - can be set to any of the window shapes supported by `getWin`, or given as a vector the same length as `number_elements` | `'Rectangular'` |

## Outputs

|  |  |
| --- | --- |
| `transducer` | k-Wave transducer object which can be used to replace the `source` or `sensor` inputs of `kspaceFirstOrder3D` |

In addition to the input parameters given above (which are also accessible after the transducer has been created), the `kWaveTransducer` object has a number of dependent properties and methods.

| Property | Description |
| --- | --- |
| `input_signal` | user defined input signal appended and prepended with additional zeros depending on the values of `focus_distance`, `elevation_focus_distance`, and `steering_angle` |
| `mask` | binary mask of the active transducer elements |
| `number_active_elements` | current number of active transducer elements |
| `transducer_width` | total width of the transducer in grid points |
|  |  |
| Method | Description |
| --- | --- |
| `active_elements_mask` | return a binary mask of the active transducer elements (identical to `mask`) |
| `all_elements_mask` | return a binary mask of all the transducer elements (both active and inactive) |
| `beamforming_delays` | return a vector of the beam forming delays (in units of time samples) for each active element based on the focus and steering angle settings |
| `combine_sensor_data(sensor_data)` | combine the sensor data returned by `kspaceFirstOrder-OMP` and `kspaceFirstOrder-CUDA` to give a single time series per active transducer element (anlogous to the output from the MATLAB code), rather than a single time series per grid point |
| `delay_mask` | return a mask of the active transducer elements, where the mask values contain the beamforming delays (an integer input can also be given to control the beamforming delays used, where 1: both delays, 2: elevation only, 3: azimuth only) |
| `elevation_beamforming_delays` | return a vector of the elevation beam forming delays (in units of time samples) for each active element based on the elevation focus setting |
| `get_receive_apodization` | return the receive apodization |
| `get_transmit_apodization` | return the transmit apodization |
| `indexed_active_elements_mask` | return a mask of the active transducer elements, where the mask values indicate which transducer element each grid point corresponds to |
| `indexed_elements_mask` | return a mask of all the transducer elements (both active and inactive), where the mask values indicate which transducer element each grid point corresponds to |
| `plot` | plot the transducer using `voxelPlot` |
| `properties` | print a list of the transducer properties to the command line |
| `scan_line(sensor_data)` | combine the input sensor data using the current apodization and beamforming setting to generate a single scan line |
| `transmit_apodization_mask` | return a mask of the active transducer elements, where the mask values contain the apodization weights |

For example, running the following commands at the command line

```
kgrid = kWaveGrid(128, 1e-3, 128, 1e-3, 128, 1e-3);
kgrid.makeTime(1500);
settings.number_elements = 72;
settings.active_elements = zeros(72, 1);
settings.active_elements(1:16) = 1;
transducer = kWaveTransducer(kgrid, settings);
transducer.properties
```

will produce the following output:

```
k-Wave Transducer Properties
  transducer position: [1  1  1]
  transducer width: 72mm (72 grid points)
  number of elements: 72
  number of active elements: 16 (elements 1 to 16)
  element width: 1mm (1 grid points)
  element spacing (kerf): 0ym (0 grid points)
  element pitch: 1mm (1 grid points)
  element length: 20mm (20 grid points)
  sound speed: 1540m/s
  focus distance: infinite
  elevation focus distance: infinite
  steering angle: 0 degrees
```

## Examples

- Defining An Ultrasound Transducer
- Simulating Ultrasound Beam Patterns
- Using An Ultrasound Transducer As A Sensor
- Simulating B-mode Ultrasound Images
- Simulating B-mode Images Using A Phased Array

## See Also

`kWaveGrid`, `kspaceFirstOrder3D`
